# Globally optimal trial design and risk sharing arrangements are key to avoiding opportunity costs of delay and enabling equitable, feasible and effective global vaccine research and implementation in current or future pandemics

**DOI:** 10.3389/fpubh.2022.1085319

**Published:** 2022-12-13

**Authors:** Simon Eckermann

**Affiliations:** School of Health and Society, University of Wollongong, Wollongong, NSW, Australia

**Keywords:** opportunity cost of delay, effective pandemic solutions, optimal global trial design, risk sharing agreements, COVID-19 mutation waves, effective and equitable global vaccination

## Abstract

Global vaccination in the face of pandemics such as COVID-19 and new variants is a race against time. Avoiding the opportunity costs of delay and the associated health, social, and downstream economic impacts is a challenge and an imperative. Failures to address the global challenges posed by COVID-19 have become increasingly evident as waves of vaccine-evading mutations have emerged, facilitated by unequal vaccination coverage and diminishing immunity against new variants worldwide. To address these challenges, societal decision-makers (governments) and industry manufacturer interests must be better aligned for rapid, globally optimal trial design, ideally with research coverage, implementation, and accessibility of effective vaccines across joint research, implementation, and distribution cycles to address pandemic evolution in real time. Value of information (VoI) methods for optimal global trial design and risk-sharing arrangements align the research, distribution, and implementation interests and efforts globally to meet head-on the imperative of avoiding opportunity costs of delay and enabling consistent global solutions with maximizing local and global net benefits. They uniquely enable feasible early adoption of the most promising strategies in real time while the best globally translatable evidence is collected and interests are aligned for global distribution and implementation. Furthermore, these methods are generally shown to be imperative for feasible, fast, and optimal solutions across joint research, reimbursement, and regulatory processes for current and future pandemics and other global existential threats. Establishing pathways for globally optimal trial designs, risk-sharing agreements, and efficient translation to practice is urgent on many fronts.

## Introduction

With rapidly evolving SARS-CoV-2 mutations and variants, vaccinating populations against the COVID-19 pandemic has been a global race against time (1, 2). The challenge has been to quickly develop and implement evidence to avoid ever more transmissible and potentially pathogenic mutations evolving and spreading from immunocompromised populations (3–6). Fast, effective, and globally equitable vaccination solutions could have prevented the spread of COVID-19 pandemic waves from the original variant B.1.1.7 (alpha) to B.1.351 (beta), P.1 (gamma), B.1.617.2 (delta), B.1.1.529 (omicron), as well as emerging mutations. Critically, their litany of associated direct and downstream global health, health system, and economic impacts (7–18).

## Health and health system impacts of the COVID-19 pandemic

Continuing COVID-19 pandemic waves have resulted in direct acute disease and mortality, as well as long-term repercussions from diminished health system access for other conditions and, more broadly, a decline in the immunity of individuals and populations (9–18). Current evidence points to immune system exhaustion and dysfunction up to 24 weeks following a COVID-19 infection and susceptibility to other diseases (11–13). Studies in the UK have found high prevalence of long COVID in those who have been hospitalized [75% at 12 months (14)], a 2% rate of long COVID symptoms for more than 4 weeks generally in the UK population during December 2021 (15) and decline in IQ from having COVID after 6 months, even in those who recover from COVID (16). More recently, according to a study, at 49 weeks post-COVID-19 infection, people exhibited significantly elevated rates of the first thrombosis, and venous thrombosis events were reported to persist from very high initial levels to still elevated levels (17).

Recent US evidence (18) shows significantly elevated risk at 12 months for neurological sequelae, including ischemic and hemorrhagic stroke, cognition and memory disorders, peripheral nervous system disorders, episodic disorders (e.g., migraine and seizures), extrapyramidal and movement disorders, mental health disorders, musculoskeletal disorders, sensory disorders, Guillain–Barré syndrome, and encephalitis or encephalopathy. Concerningly, these higher risks are present in both hospitalized and non-hospitalized post-acute individuals who survive COVID-19 for more than 30 days.

Uncertainty persists over the duration of COVID-19's effects on the immune system. It is evident that having a strong immune system and being vaccinated (with effective vaccinations for all current variations) are essential for having the best chance of preventing and combating any sickness, both individually and for populations locally or globally. The opportunity costs of delaying effective vaccine protection during a pandemic locally include a loss in net benefits associated with the failure to stop the pandemic spread and downstream health, health system, and associated economic impacts (7–18). Globally, particularly during the COVID-19 pandemic, they also include the failure to prevent new waves from new variant mutations arising in immunocompromised populations (1–6, 19–21).

## Vaccine protection against prior, current, emerging, and future variants

First-generation mRNA vaccines were effective in immunizing against initial COVID-19 variants in 2020 (alpha, beta, and gamma) and remain relatively effective with mutations of variants from 2021 and early 2022 (Delta and Omicron) in preventing hospitalization and deaths but not infection (1, 3, 22, 23). Nevertheless, immunization against new variants and mutations of concern emerging from immunocompromised populations has been confounded by vaccine-evading mutations and rapidly decreasing infection protection of booster vaccines from original variants (3, 19–21). Our immune systems' memory imprinting may also prevent significant advantages from simply exchanging new for old variants in mRNA vaccines (24–26).

The benefits of new variant-based vaccines, boosters, and related research grow stronger when pre-existing immunity and/or effectiveness of current treatment are suddenly lowered by the appearance of new mutations or variant/s (26). Most recently, the fast-emerging “scrabble” variants (BQ.1, BQ.1.1, and BA.4.6) have evolved resistance to monoclonal antibody therapies, while antivirals (e.g., Paxlovid and Remdesivir) currently still provide effective treatment for those who can take them (27). Immunologists indicate more effective vaccines for emerging and future variants will need appropriate coverage across previous and current variants and mutations and be adaptable to new evidence and variant evolution (24, 25).

## Bottom line challenge for future global population pandemic protection

Given that rapid mutations arise and proliferate in the most immunocompromised populations (3–6), the global population's immune system needs to be supported to overcome COVID-19 pandemic waves and rolling mauls of continuing health and economic impacts globally.

The most recent WHO policy has issued an urgent call for governments around the world to examine their vaccination policies and strengthen them in preparation for ongoing COVID-19 mutations and future pandemics. This article focuses on WHO vaccination development, distribution, and population delivery goals (10). In the following sections, I will show that these goals are jointly, feasibly, and systematically addressed globally in processes from research through distribution and implementation in practice with value of information (VoI) methods for globally optimal trial design (28) and risk-sharing agreements (29).

## VoI methods for global vaccine solutions?

Making decisions with uncertain relative net benefit across potentially optimal alternative strategies, VoI methods inform optimal decision making and trial design. They compare the expected value (expected reduced probability of and payoffs from bad decisions or reduced expected loss of perfect information) relative to the expected cost (direct and opportunity cost) of research (30–33). Importantly, in doing so, they should account for key decision contexts for decision-making (34, 35). Those contexts include the time and opportunity costs of delay associated with delaying and trialing (33, 36, 37), the feasibility of adopting and trialing (29), and related considerations of whether decisions are local or global (28). Furthermore, the degree of implementation and pricing of new technologies under conditions of uncertainty is conditional on the strength of evidence (29, 38, 39).

**Figure 1:**
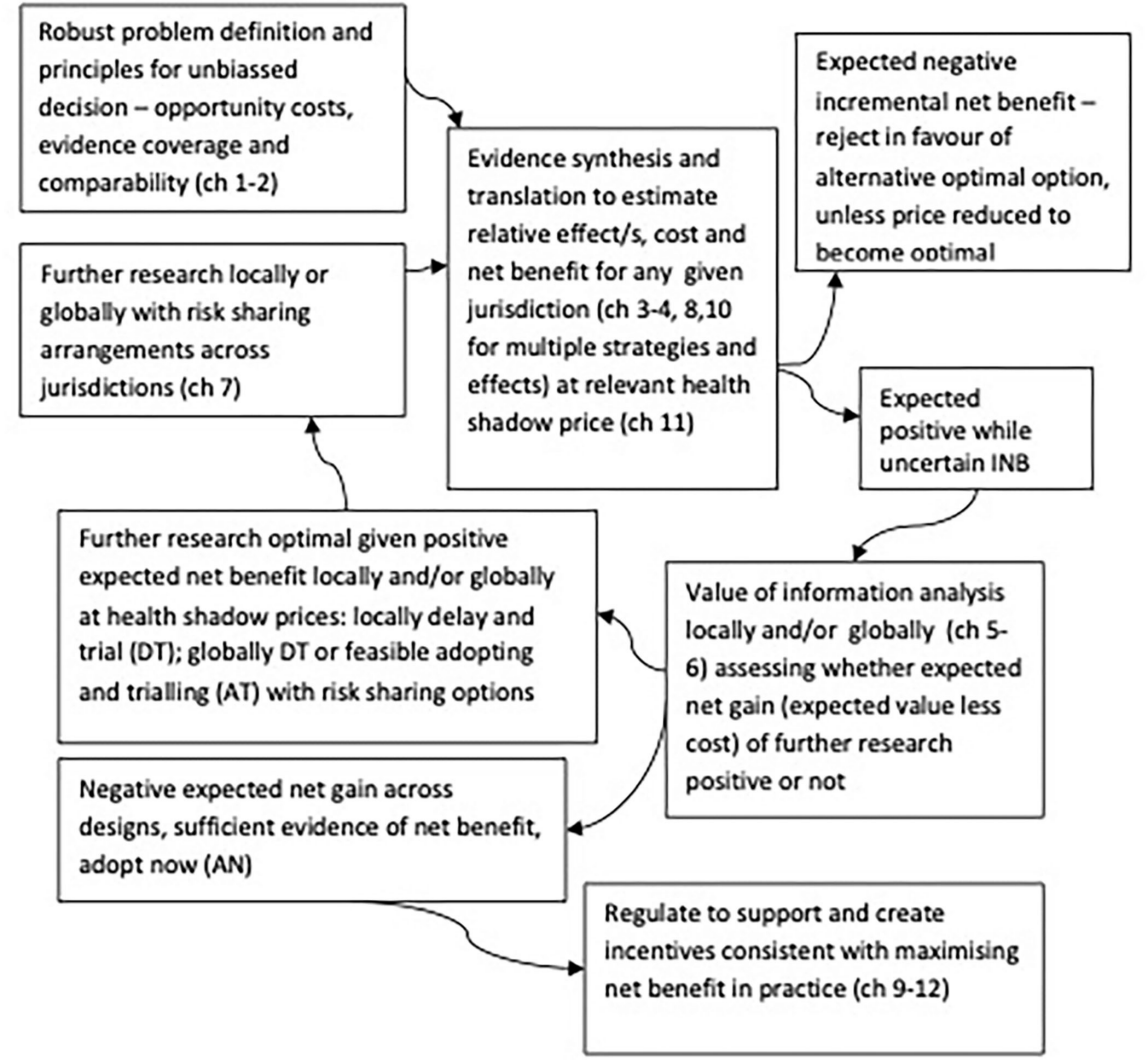
Optimal decision making cycles for joint research, reimbursement, regulation and practice locally and globally, adapted from Eckermann (34).

Applying VoI methods with appropriate consideration of those contexts systematically enables optimal joint research, adoption, and regulatory investment decisions consistent with maximizing net benefits locally and globally (34, 35), as shown in Figure 1. Figure 1 highlights that VoI methods are key to systematically synthesizing evidence and divining optimal decision-making across joint research, adoption, and regulatory implementation decisions. Specifically, identifying efficient and optimal pathways for whether to adopt or reject any strategy now based on current evidence (current evidence is sufficient) or whether further research is optimal locally or globally (where valuable options to feasibly adopt and trial, and robustly risk share arise) (28, 29).

## Moving beyond optimal local trial design

In the application of VoI methods before Eckermann and Willan (28), while the synthesis of global evidence-informed prior distributions for a relative net benefit of alternatives in any jurisdiction was considered, the prospective value of such trials was only considered locally within that jurisdiction. Thus, evidence from outside the jurisdiction had retrospective value; however, only evidence from within the jurisdiction was assumed to have prospective value (28). That said, publicly available evidence from trials is a non-rival and non-excludable public good. Indeed, the freely available evidence is now explicitly supported by the US Office of Science and Technology Policy's (OSTP) announcement on 25 August 2022: “to make the results of federally funded scientific research in the United States immediately free to access and available to all” (40).

Hence, provided trial evidence can be translated, new evidence arising in one jurisdiction is expected to have value in another. Consequently, where prospective VoI from trials across jurisdictions is appropriately considered, additional viable options include using side payments to influence trial design, avoiding fixed trial costs, recognizing the joint value of research, and increasing the strength and implementation of evidence with a large definitive trial. Hence, a combined optimal trial across two jurisdictions improves on separate optimal trials within each jurisdiction. Eckermann and Willan (28) extend that principle across all jurisdictions to address the question: What is the globally optimal trial design?

## Globally optimal trial design

Under the non-rival and non-excludable characteristics of a public good, the expected value of a global trial can be summed across jurisdictions. That global value less cost of the trial or global expected net gain (ENG) can then be optimized. Each jurisdiction can make optimal local decisions about whether to adopt or delay a trial as part of a globally optimal trial designed to optimize and share greater global ENG than locally optimal solutions. Notably, a key source of those gains is that global optimal VOI trials overcome the infeasibility of jurisdictions, faced with positive while uncertain INB, to adopt and trial (AT) within the jurisdiction, given the inability to recruit informed patients where they have certainty of new therapy outside the trial setting (28, 29). Global VoI trials make it feasible for jurisdictions to AT while trial patients are recruited in jurisdictions that delay and trial (DT).

Each jurisdiction can identify whether to optimally adopt or delay and trial (AT or DT) given their prior distribution for INB and local ENG. That is, given their local EVSI of trial dependent on Cr for AT and local EVSI less opportunity cost for DT (28). As Eckermann and Willan (28) show, the globally optimal trial design given optimal local decision-making is given by the set of trial recruitment choices across jurisdictions (n_*j*_ per arm) and hence the global trial size n per arm as a sum of the njs that maximize the global expected value less trial costs: $\overset{J}{\underset{j=1}{\sum }}\max\left(oENG_{Dj}\left(n,n_{j}\right),oENG_{Aj}\left(n,n_{j}\right)\right)\;-\overset{J}{\underset{j=1}{\sum }}\left(C_{fj}+2n_{j}C_{vj}\right).\;$

Each jurisdiction chooses to delay or adopt to maximize local ENG (excluding direct trial costs). Direct costs (fixed *C*_*fj*_ and variable *C*_*vj*_) of trials incurred locally are shared globally as part of the trial design with globally pooled side payments to ensure optimal DT as part of Pareto optimal and equitable solutions (28). Where evidence is translatable across jurisdictions where evidence is translatable across jurisdictions there is always scope for such arrangements given globally optimal solutions are better (pareto optimal with higher ENG globally) than those locally. That is particularly for pandemics, given feasible AT is necessary to avoid opportunity costs of delay. Indeed, options to feasibly AT as part of globally optimal trial design and benefit from risk sharing agreements (28, 29) would be critical for jurisdictions who faced higher risks and reduced or no capacity to recruit patients for research during COVID-19 pandemic waves (e.g. Peru, Brazil, Mexico).

## Global trials and risk sharing

Risk sharing relies on continuing evidence collection to support meaningful contracts for future contingencies. Risk sharing locally is inherently incomplete, because an inability to AT within a jurisdiction implies only observational evidence from practice is available. Evidence from one arm or from selected patients results in incomplete contracts in assessing relative effects or net benefit (NB) over time. However, a global trial supports feasible AT and robust evidence of relative effects and NB across treatments to support meaningful specification of future contingencies. Consequently, global trials uniquely enable robust risk sharing (29).

From a manufacturer's perspective, adopting while simultaneously conducting a global trial and risk sharing offers advantages over delaying and trialing, as it allows a revenue stream while gaining additional evidence. Additional evidence is also *a priori* expected to increase the strength of evidence and the future degree of implementation (37). Hence, optimally designed global trials effectively act as a circuit breaker in enabling feasible AT with earlier access and globally translatable evidence. AT is preferred over DT for both manufacturers and decision-makers alike. Risk-sharing arrangements can also mitigate the impacts of reversal costs with AT by making pricing conditional on the strength of evidence for INB.

The potential for increased bias with manufacturer trials (42–45) is mitigated with global VoI trials because they inherently design for global translation of evidence across jurisdictions, in return for which, companies have earlier adoption (28, 29). Hence, societal decision makers are in a strong position to ensure potential for bias are avoided as global gate-holders to permitting adopting and trialling (AT). Indeed, avoiding biases is inherent in globally optimal trial design and risk sharing arrangements.

## Key advantages of a globally optimal trial design for pandemic management from research to global translation and practice

The advantages of optimal global VoI trial design (28) and risk-sharing arrangements (29) across WHO joint vaccination development, distribution, and delivery goals (10) can be summarized as follows:

- Recognizing the higher global VoI in optimal trial design with freely available public data, supported by and supporting the value of OSTP guidance (40) and implications of that guidance for free research and publishing models (45);
- Minimizing sampling costs (fixed, variable, and opportunity costs) in allocating samples across jurisdictions (28);
- Avoiding cherry picking and reducing heterogeneity and associated “Frankenstein's Monster” effects arising with multiple trials in evidence synthesis (41);
- Overcoming market failure from free rider effects (no or too small trials) and sub-optimal spreading of fixed costs (too many trials) (28);
- Stronger global evidence is generated to inform regulators and improve implementation within and across jurisdictions (28, 29); and
- Enabling feasible and robust risk-sharing arrangements globally to better align societal decision-maker interests and manufacturer interests in avoiding opportunity costs of delay for jurisdictions who AT as part of the global trial (29).

Collectively, those advantages are key to addressing COVID-19 vaccination challenges and finding feasible and optimal global solutions for pandemics or similar global problems (e.g., global warming):

- With significant opportunity costs of delay;
- Needing the best and most translatable global evidence; and
- Requiring global risk-sharing arrangements to reinforce alignment across industries and governments for fast global distribution and the implementation of optimal strategies.

Proposed methods generate the best globally translatable evidence and avoid opportunity costs of a delay from research to global translation, distribution, and implementation in practice (28, 29, 34).

## Key advantages with vaccine science now—Choosing which needle/s in which haystack/s

Global evidence and translation covering different vaccine variant combinations are required both currently and in real time for future mutations, given immunity imprinting and likely need for vaccines targeting only novel spikes in new variants (25–27, 46). Therefore, globally optimal trial designs and risk-sharing agreements are ideal with the ability to adapt and trial (with vaccination in all arms), quickly expand coverage globally and avoid opportunity costs of delay and evidence in real time for potential emerging new variants. Critically, those trials provide the best research evidence globally and the best coverage and translation to uptake and implementation in practice. They mutually and globally support designs such as the COVAIL trial, evaluating the immune responses of candidate SARS-CoV-2 variant vaccines, alone or in combination (47).

## Discussion: VoI methods and pandemic vaccination response

Globally optimal trial design and risk-sharing agreements align with the research and implementation interests and efforts of countries and vaccine manufacturers globally for doing what is necessary to combat the pandemic and avoiding opportunity costs of delay across all countries while obtaining the best global evidence. Fast, effective global vaccine coverage is key to building global immunity and preventing new variants and their health, social, and economic impacts, especially given that new mutations and variants develop and thrive in immunocompromised populations and are quickly spread in the globally connected world and economies of the 21st century (1, 2).

With new-generation boosters, the global experience with the first-world development of low-temperature first-generation vaccinations, highly inequitable manufacture, distribution, and administration (9, 10, 48, 49), and political straw-man marketing of performance (50) risks being repeated. Rather than addressing the key challenge of global immunity coverage, vaccination efforts have been fragmented by national and multinational vested interests. They continue to falter in their attempt to vaccinate people against current variants in the context of fast-evolving and increasingly vaccine-evading mutations (1, 2, 10, 25–27, 46, 48–50). Furthermore, those failures are compounded by overstretched health systems in all countries, which have struggled to cope with the short-term and acute effects of the pandemic spread, let alone long-term COVID or the emergence and re-emergence of other diseases in immunocompromised populations (3–9).

Addressing those global challenges requires a better alignment of societal decision-makers' (government) and industry's interests in vaccine trial design and their global implementation, distribution, and access across joint research, implementation, investment, and regulatory cycles [Figure 1 (34)]. Appropriate global translation in coverage is inherent with optimal global VoI trial design and risk sharing as proposed (28, 29), enabling feasible, fast, and effective solutions with appropriate global coverage that prevent the opportunity cost of delay. VoI methods have just started to be applied to consider COVID-19 treatment therapies (51). Prevention of new pandemic waves (and further impacts of prior and emerging variants and mutations) has direct and downstream health, health system, and wider social and economic cost implications (7–18). As a result, the application of global optimal trial design and risk sharing (28, 29) for vaccination strategies is more urgent and has a much greater global expected value (28–33) and return on investment (34, 35) to societal decision-making worldwide.

## Research extensions

A question that naturally arises is, “How far can the framework for optimal global trial design and risk-sharing arrangements be extended?” This question has been previously addressed generally for optimal joint research, reimbursement, and regulatory decision-making (34, 35). Nevertheless, some points are key in reference to global problems such as the COVID-19 pandemic. While this article has focused on optimal trial design and risk-sharing agreements (28, 29), it is important to note the following:

- By lowering the opportunity cost of delay for globally translatable evidence into practice, these strategies aim to maximize global community net benefit and align societal decision-making (governments) and industry (manufacturers) interests toward that objective;
- In mutually supporting the globally optimal trial design and risk-sharing arrangements, the net benefit correspondence theorem (34, 52–56) uniquely provides a robust mechanism for effective and efficient translation of net benefit evidence in comparison to multiple strategies (34, 52, 54) as well as multiple outcomes or practices (34, 53, 55, 56), which are key for accountable evidence translation, implementation, and regulation.

## Conclusion

No country is safe or immune from the COVID-19 pandemic until new variants of concern are prevented from arising in immunocompromised populations; we have become globally immune to recent variants. As Fontanet (1) summarized, “…the end of the pandemic is only possible when vaccines that are effective against circulating variants are distributed equitably across the world.”

This article has highlighted that optimal global trial design (28) and risk-sharing arrangements (29) provide the necessary methods for systematically properly addressing these challenges, enabling the best global research and its efficient and equitable distribution and implementation. Importantly, these previously developed methods for optimal global VOI trial design and risk-sharing arrangements provide the only feasible and ideal means for that to occur.

## Data availability statement

The original contributions presented in the study are included in the article/supplementary material, further inquiries can be directed to the corresponding author.

## Author contributions

The author confirms being the sole contributor of this work and has approved it for publication.
